# Chitin-Lignin Material as a Novel Matrix for Enzyme Immobilization

**DOI:** 10.3390/md13042424

**Published:** 2015-04-20

**Authors:** Jakub Zdarta, Łukasz Klapiszewski, Marcin Wysokowski, Małgorzata Norman, Agnieszka Kołodziejczak-Radzimska, Dariusz Moszyński, Hermann Ehrlich, Hieronim Maciejewski, Allison L. Stelling, Teofil Jesionowski

**Affiliations:** 1 Institute of Chemical Technology and Engineering, Faculty of Chemical Technology, Poznan University of Technology, Berdychowo 4, 60965 Poznan, Poland; E-Mails: jakub_zdarta@wp.pl (J.Z.); lukasz.klapiszewski@put.poznan.pl (L.K.); wysokowski@wp.pl (M.W.); malgorzata.norman@hotmail.com (M.N.); agnieszka.kolodziejczak-radzimska@put.poznan.pl (A.K.-R.); 2 Institute of Inorganic Chemical Technology and Environmental Engineering, West Pomeranian University of Technology, Pulaskiego 10, 70322 Szczecin, Poland; E-Mail: dmoszynski@zut.edu.pl; 3 Institute of Experimental Physics, TU Bergakademie Freiberg, Leipziger Str. 23, 09599 Freiberg, Germany; E-Mail: Hermann.Ehrlich@physik.tu-freiberg.de; 4 Adam Mickiewicz University in Poznan, Faculty of Chemistry, Umultowska 89b, 61614 Poznan, Poland; E-Mail: maciejm@amu.edu.pl; 5 Poznan Science and Technology Park, Adam Mickiewicz University Fundation, Rubież 46, 61612 Poznan, Poland; 6 Duke University, Center for Materials Genomics, Department of Mechanical Engineering and Materials Science,144 Hudson Hall, Durham, NC 27708, USA; E-Mail: antistokes@gmail.com

**Keywords:** chitin-lignin matrix, enzyme immobilization, hydrolytic activity, lipase, immobilized lipase stability

## Abstract

Innovative materials were made via the combination of chitin and lignin, and the immobilization of lipase from *Aspergillus niger*. Analysis by techniques including FTIR, XPS and ^13^C CP MAS NMR confirmed the effective immobilization of the enzyme on the surface of the composite support. The electrokinetic properties of the resulting systems were also determined. Results obtained from elemental analysis and by the Bradford method enabled the determination of optimum parameters for the immobilization process. Based on the hydrolysis reaction of para-nitrophenyl palmitate, a determination was made of the catalytic activity, thermal and pH stability, and reusability. The systems with immobilized enzymes were found to have a hydrolytic activity of 5.72 mU, and increased thermal and pH stability compared with the native lipase. The products were also shown to retain approximately 80% of their initial catalytic activity, even after 20 reaction cycles. The immobilization process, using a cheap, non-toxic matrix of natural origin, leads to systems with potential applications in wastewater remediation processes and in biosensors.

## 1. Introduction

Continuing technological progress means that scientists are constantly finding new solutions that make use of lignin and its derivatives. When suitably modified, lignin is a polarographically active material [1] capable of undergoing a variety of electrochemical reactions, in the course of both oxidation and reduction [2]. Consequently, in recent years it has found interesting applications in electrochemistry. One of these was the creation of a cheap and fully environmentally friendly cathode, developed by Milczarek and Inganäs [3]. The valuable properties of lignin and its particular structure had previously been exploited by Milczarek in the construction of electrochemical sensors and detectors, as described in [4,5,6,7]. Interesting work using lignin-based material to create an innovative, cheap battery was reported by Gnedenkov *et al.* [8,9,10]. Literature reports also indicate the possibility of using lignocellulose materials, including pure lignin, as a filler in a wide range of polymers, both in strongly polar (poly(ethylene terephthalate)—PET; poly(ethylene oxide)—PEO) [11,12] and in hydrophobic (polypropylene—PP) [13,14] polymer matrices. Studies have also been carried out using poly(vinyl chloride) [15]. The biopolymer may also serve as a potential cheap and easily available biosorbent for environmentally harmful metal ions [16,17,18,19,20]. As a sorbent, lignin may be obtained chiefly as a waste product of the paper industry, and subjected to chemical modification to increase the number of functional groups [21,22]. It has also been reported that lignin has multifunctional barrier properties, protecting against harmful UV radiation, as well as antibacterial properties [23]. There are also promising possibilities for the use of lignin in the pharmaceutical industry and in medicine.

Chitin is an aminopolysaccharide, built of a long polymer chain consisting of *N*-acetylglucosamine units connected by β-1,4-glycoside bonds [24]. Chitin is a natural polymer, obtained chiefly from the shells of marine invertebrates, including the marine sponges [25,26,27,28]. It is friendly to the natural environment, and it exhibits high chemical stability and high reactivity, and is also non-toxic, bioactive, biodegradable and biocompatible [29]. Because of these features it is used in many areas of biomedicine and biotechnology [30,31]. One of these fields is the immobilization of enzymes [32,33,34]. Krajewska [35] presents a wide-ranging review of the literature concerning the use of chitin as a support for many catalytic proteins. Enzymes were immobilized by cross-linking with chitin by glutaraldehyde to reduce the viscosity of fruit and vegetable juices [36]. Outside the food industry, mention might be made of the use of enzymes immobilized on chitin via physisorption [37] or with the formation of covalent bonds [38] to detect and remove phenols. One of the most industrially useful groups of enzymes are the lipases, which are hydrophobic enzymes. To take full advantage of their technical and economic possibilities, they are used in a form immobilized on chitin [39]. An important factor in the widespread use of chitin as a support is the universality of the forms in which it can be used. Available morphological forms include powder, flakes, beads, nanoscale whiskers and fibers [40].

The creation of a stable material with defined properties provides the possibility of combining the undoubted advantages of both precursors, such as the aforementioned biocompatibility and non-toxicity, in the process of enzyme immobilization. The presence of multiple reactive functional groups in the structure of both materials increases their affinity to biomolecules [41]. It should be noted that the fact that the matrix is made using relatively cheap waste materials has a positive impact with regard to the economic aspects of the immobilization process [42]. The systems so produced may have potential uses in many fields where there is a need for highly pure and non-toxic catalysts.

The aim of the present study was to use a chitin-lignin material as a novel matrix for immobilization by adsorption of the lipase from *Aspergillus niger*. This is work of an innovative aspect, because there are no reports in the literature concerning the use of this system in enzyme immobilization. The systems produced may find uses in the transesterification and hydrolysis of a wide range of compounds, as well as in the production of biosensors. The results of the analysis confirmed the effective immobilization of the lipase on the chitin-lignin support. A detailed analysis was also made of the effect of process parameters on the properties of the resulting systems, and it was shown that lipase immobilized on the composite offers greater thermal and chemical stability than the native enzyme.

## 2. Results and Discussion

### 2.1. Physicochemical Evaluation

#### 2.1.1. FTIR Spectroscopy

**Figure 1:**
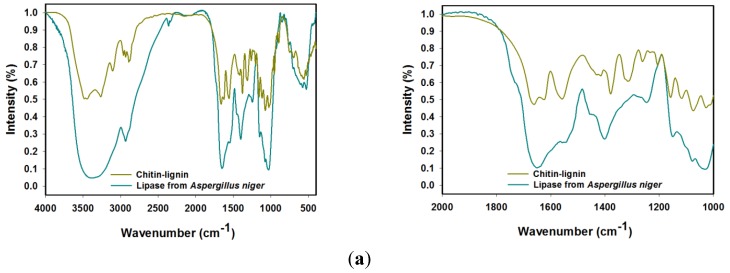

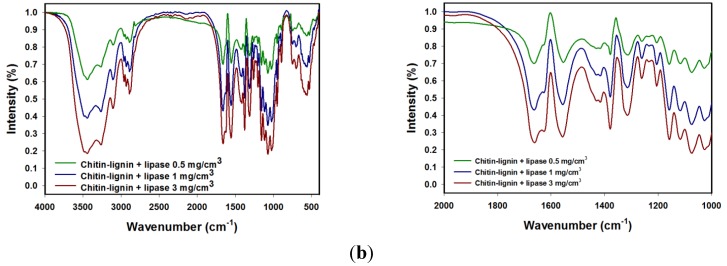
FTIR spectra of chitin-lignin composite and lipase (**a**) and selected products following 24 h of enzyme immobilization (**b**), in two different spectral range.

Figure 1 shows the FTIR spectra of the chitin–lignin material, lipase from *Aspergillus niger* (Figure 1a), and the products following enzyme immobilization (Figure 1b). The major bands are summarized in Table 1.

**Table 1:** Maximal vibrational wavenumbers (cm^−1^) attributed to lipase from *Aspergillus niger*, chitin-lignin material, and products following immobilization.

Lipase from *Aspergillus niger*	Chitin-Lignin Material	Products after Immobilization	Vibrational Assignment
3460	3444	3457	O-H stretching
3242	3257	3264	N-H stretching
-	3111	3112	C_Ar_-H stretching
2931	2965, 2930, 2877	2966, 2935, 2879	CH_x_ stretching
-	1674	1676	C=O stretching
1647	1625	1639	amide I stretching
1546	1556	1552	amide II bending
1448	1432	1438	CH_2_ bending
-	1420	1417	C_Ar_-C_Ar_ stretching
1402	1388	1401	O–H stretching
-	1323	1329	C-O (syringyl unit) streching
1257	1268	1261	amide III bending
1151, 1073, 1037	1158, 1116, 1077, 1022	1162, 1113, 1081, 1027	C-O-C (ring), C-O stretching
-	953	957	CH_3_ bending
-	903	905	β-1,4-glycosidic bonds
-	745	745	aromatic C-H(guaiacyl unit), bending
576	558	571	N-H bending
531	527	530	C-C scissoring

Analysis of the FTIR spectrum of the enzyme prior to immobilization shows the presence of a band in the range 3550–3200 cm^−1^ associated with stretching vibrations of O-H and N-H groups, and one at wavenumber 2931 cm^−1^ from stretching vibrations of C-H (CH_3_ and CH_2_). The most important signals in the spectrum of the native lipase are peaks at wavenumbers 1647 cm^−1^, 1546 cm^−1^ and 1257 cm^−1^, whose presence is characteristic of stretching vibrations of amide I, II and III bonds [43,44]. The FTIR spectrum of the enzyme also features a peak at wavenumber 1402 cm^−1^, generated by stretching vibrations of O-H groups, and a low-intensity signal at 1448 cm^−1^ confirming the presence of bending vibrations of CH_2_. The group of signals at 1151 cm^−1^, 1073 cm^−1^ and 1037 cm^−1^ are associated with the presence of C-O-C bonds in the protein structure [45]. In addition, of note are two signals below 1000 cm^−1^: at 576 cm^−1^ a band of N-H stretching vibrations, and at 531 cm^−1^ a band of scissor vibrations of the C-C bonds forming the skeleton of the enzyme structure [46].

Analysis of the spectrum of the chitin–lignin matrix confirms that the expected product was obtained. It also features a large number of bands, this being a result of the complex structure of the system. Attention is drawn to the bands with maxima at 3444 cm^−1^ and 3257 cm^−1^, attributed to stretching vibrations of O-H and N-H groups. A peak with a maximum at 3111 cm^−1^ is associated with stretching vibrations of C_Ar_-H groups present in the lignin structure [47]. A series of signals in the range 2970–2870 cm^−1^ confirms the presence of CH_2_ and CH_3_ groups in the structure of the composite, while the distinct band with a maximum at 1674 cm^−1^ comes from stretching vibrations of C=O bonds. Four signals between 1160 cm^−1^ and 1020 cm^−1^ can be attributed to stretching vibrations of C-O-C bonds in the glucose ring in chitin, as well as other C-O bonds in the material [48]. The interpretation of the carbon–oxygen bonds present in the system is supplemented by a peak at wavenumber 905 cm^−1^, which is a consequence of the β-1,4-glycosidic bonds in chitin [49]. Note should also be taken of the signals originating from vibrations of amide I, II and III bonds. These are bands analogous to those present in the enzyme structure, but appearing at slightly different wavenumbers, respectively 1639 cm^−1^, 1552 cm^−1^ and 1261 cm^−1^, as a result of the different chemical environment of the bonds. Very significant bands, confirming the production of a chitin–lignin material, are present at 1420 cm^−1^, 1329 cm^−1^ and 745 cm^−1^, and originate from the stretching and bending vibrations of the aromatic structures present in lignin [50].

The FTIR spectra of the systems following immobilization carried out for 24 h using solutions of the enzyme in various concentrations are shown in Figure 1b. Analysis of the data obtained shows that the lipase was effectively immobilized on the matrix surface. In spite of the similarity of the bands present on the spectra of the support and the enzyme, an indication is provided by the presence of signals associated with vibrations of amide I, II and III bonds contained in the protein structure, at wavenumbers 1639 cm^−1^, 1552 cm^−1^ and 1261 cm^−1^ respectively [51]. The intensity of these bands increases, and their absorption maxima are shifted, compared with the spectrum of the support. Analogous observations apply to the signals from stretching vibrations of O-H groups at wavenumber 3457 cm^−1^, and from stretching vibrations of C=O bonds at 1676 cm^−1^. The changes provide additional evidence confirming the immobilization, as well as indicating hydrogen bonding between the matrix and enzyme [52]. It is also interesting that as the concentration of the enzyme solution used for immobilization increases, particular bands in the product spectra become more intense. This provides indirect evidence that there is also an increase in the quantity of the enzyme deposited on the matrix surface.

#### 2.1.2. ^13^C CP MAS NMR Spectroscopy

**Figure 2:**
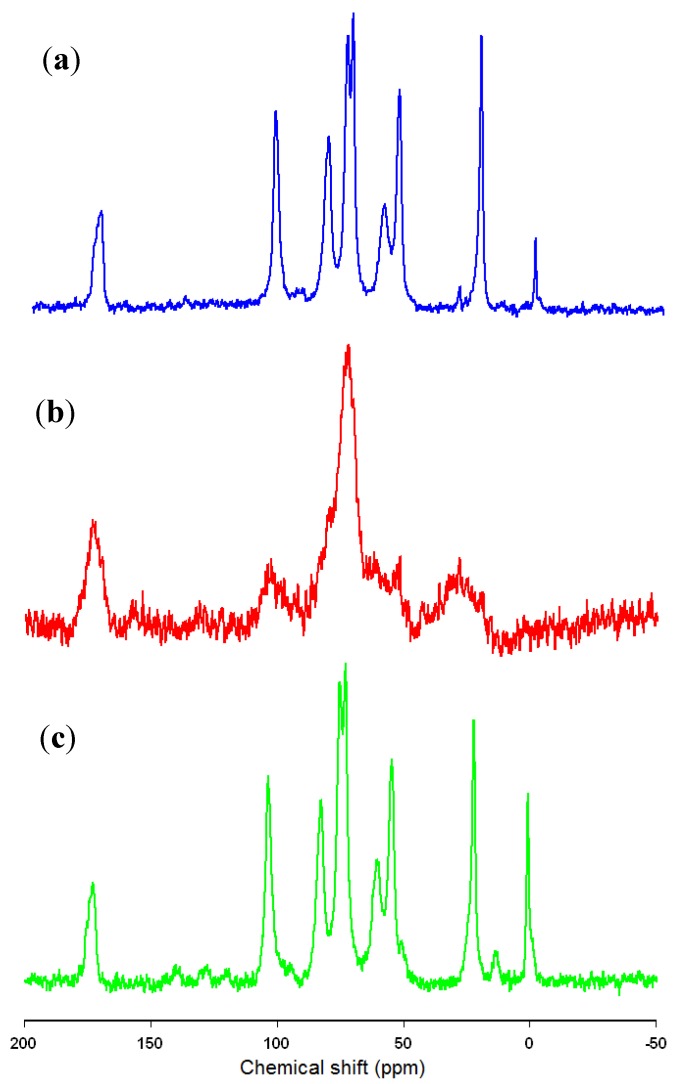
^13^C CP MAS NMR spectra of chitin-lignin (**a**); lipase (**b**) and chitin-lignin matrix with immobilized enzyme (**c**).

Figure 2 shows the ^13^C CP MAS NMR spectra of the obtained chitin–lignin material, the native lipase, and the product following 24 h of immobilization of the enzyme from solution at a concentration of 3 mg/cm^3^.

The ^13^C CP MAS NMR spectrum of the chitin-lignin material shows the presence of signals characteristic of the precursors, which provides confirmation of the effective formation of the expected material. The signal at 22 ppm originates from the carbon in CH_3_ in acetamide groups from chitin, while the entire group of peaks in the range 55–105 ppm is generated by carbon atoms in *N*-acetylglucosamine mers [53]. The distinct signal at 175 ppm originates from the carbonyl carbons in acetamide groups in the chitin structure [54]. The spectrum of the immobilized enzyme provides confirmation of the previous findings concerning the great similarity in structure of the lipase and the chitin; which is the chief component of the composite. The spectrum of the protein contains two clear signals, with maxima at 76 and 177 ppm, as well as several bands of much smaller intensity and wider range. The spectrum of the product formed after immobilization, in view of the similarity of the spectra of the precursors, does not show many changes. There is a different shape, particularly at the base, in the signals at 56 and 107 ppm. There is also a characteristic area between 115 and 145 ppm, where there appear signals which were not observed in the spectrum of the support, but which appear with low intensity in the spectrum of the native enzyme. Analysis of the ^13^C CP MAS NMR spectra confirms the effectiveness of the immobilization process and the immobilization of the enzyme on the surface of the chitin-lignin matrix. In addition, in the case of the signals on the spectrum of the system after immobilization, there is seen to be a small shift in their maxima, which may suggest that the protein is attached to the support by way of the formation of hydrogen bonds.

#### 2.1.3. Elemental Analysis

Table 2 contains the results of elemental analysis, describing the change in the content of such elements as nitrogen, carbon, hydrogen and sulfur in the immobilized enzyme preparations and in the matrix used.

**Table 2:** Elemental content of examined elements in the chitin-lignin matrix and in products following immobilization.

Enzyme Solution Concentration (mg/cm^3^)	Immobilization Time	Elemental Content (%)			
		N	C	H	S
Chitin-lignin matrix		5.07	33.86	4.93	0.03
0.5	1 min	5.23	35.42	5.40	0.02
	2 h	5.58	37.17	5.67	0.01
	24 h	6.41	37.77	5.73	0.03
1.0	1 min	5.75	38.31	5.54	0.01
	2 h	5.96	38.77	5.78	0.03
	24 h	6.66	39.81	5.95	0.02
3.0	1 min	5.96	39.01	5.91	0.03
	2 h	6.03	39.30	6.05	0.02
	24 h	6.77	39.92	6.07	0.02

The initial matrix, prior to enzyme immobilization, has a carbon content of 33.86% and a hydrogen content of 4.93%. These elements are present in the structure of both lignin and chitin. Nitrogen, found in the elemental composition of the hybrid material with a content of 5.07%, is associated with the presence of *N*-acetylglucosamine groups in chitin. The presence of sulfur in the composite is explained by the use of sulfuric acid in the kraft process used to produce the lignin precursor.

The elemental analysis of systems resulting from the immobilization of lipase on the surface of the chitin-lignin matrix showed an increase in the contents of carbon, nitrogen and hydrogen, compared with the initial material. These changes are a result of the presence of those three elements in the structure of the enzyme, and confirm the effective immobilization of the protein on the surface of the support. The increase in the content of the analyzed components with higher initial concentration of protein solution and longer time of immobilization indicates that both of these parameters have a significant effect on the quantity of enzyme immobilized. The most distinct changes compared with the chitin–lignin material were observed for the system produced following a process lasting 24 h using a solution of concentration 3 mg/cm^3^, which may be taken as confirmation that the greatest quantity of protein was immobilized under such conditions.

#### 2.1.4. XPS Analysis

The surface composition for samples of lipase, chitin–lignin material and the product following enzyme immobilization was examined with X-ray photoelectron spectroscopy. The surface of all samples is composed of carbon, oxygen and nitrogen. Some traces of calcium, potassium and sulfur were detected, but these are not considered in the quantitative calculations. The elemental surface compositions calculated from XPS data are given in Table 3.

**Table 3:** Elemental composition of the surface of samples.

Sample Name	Atomic %			N/C Ratio	O/C Ratio
	C	O	N	H	S
Lipase	58.2	30.7	11.1	0.19	0.53
Chitin-lignin matrix	61.4	32.6	6.0	0.10	0.53
Chitin-lignin + lipase	62.5	30.0	7.5	0.12	0.48

The elemental composition of the lipase as reported by Tomizuka *et al.* and expressed as a C:O:N molar ratio is 61:25:14 [55]. These values are in good agreement with the ratio obtained in the present study for the surface of lipase, namely 58:31:11. Similar good agreement is obtained for the surface composition of the chitin-lignin matrix, which was reported previously [53]. The oxygen-carbon ratio close to 0.5 obtained for chitin-lignin, as well as the surface composition of the matrix, are very close to the values observed for nanocrystalline chitin [56]. Since the elemental composition of lignin differs significantly from the ratio observed here, it is concluded that the surface of the support matrix is composed mainly of chitin. The nitrogen-carbon ratio is almost twice as high for the lipase as for the chitin-lignin material. Therefore an increase in this parameter can be used as an indicator for successful enzyme immobilization, as reported previously [57]. Indeed the N/C ratio increases from 0.10 for the pure chitin-lignin matrix to 0.12 for the sample after immobilization. The elemental analysis of samples before and after immobilization, as described in Section 2.1.3., indicates an increase of approximately 20% in the nitrogen content after enzyme immobilization. This is corroborated by XPS data. This increase in nitrogen concentration following the immobilization process is taken as indirect evidence of successful lipase immobilization.

**Figure 3:**
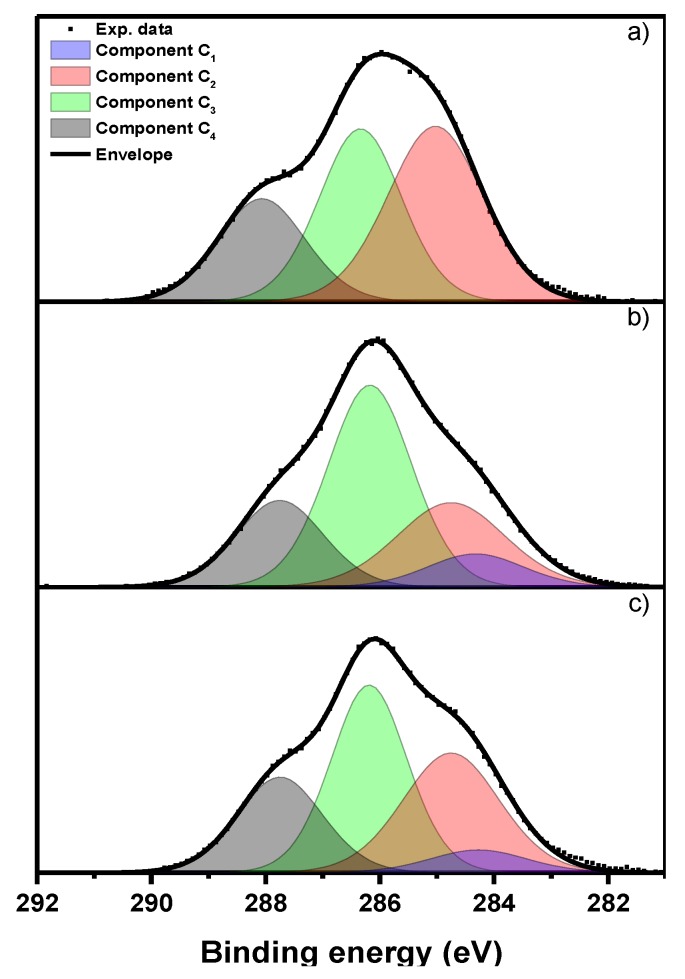
The XPS C 1s spectra for chitin-lignin (**a**); lipase (**b**); and the chitin-lignin + lipase product (**c**).

Evaluation of the chemical composition of the surface of the examined materials is based mainly on analysis of the XPS C 1s peak. The spectra have a relatively complex profile (Figure 3). Deconvolution of the experimental data was performed using a model consisting of four basic components of the C 1s transition: C_1_–C_4_. Component C_1_, with a binding energy of 284.4 ± 0.1 eV, corresponds essentially to non-functionalized carbon atoms located in the aromatic rings expected to be in the lignin structure. Component C_2_, with a binding energy of 284.8 eV, is attributed to all other non-functionalized sp^2^ and sp^3^ carbon atoms, bonded either to other carbon or to hydrogen atoms. Component C_3_, shifted by 1.4 ± 0.2 eV from component C_2_ in the direction of increasing binding energies, is attributed to a set of groups with a carbon atom bonded to one atom of oxygen or nitrogen. These include the following functional groups which are presumed to be present in the studied materials: C-O-C, C-OH, C-N-C, C-NH_2_. Component C_4_, shifted by 2.9 ± 0.2 eV from component C_2_ in the direction of increasing binding energies, also corresponds to a set of functional groups: C=O, O-C-O, N-C-O and N-C=O. The binding energy interpretations given above are based on the energy shifts given in Appendix E [58]. A relative surface functional group composition obtained from decomposition of the C 1s signal is given in Table 4. The total C 1s peak intensity is taken as 100.

**Table 4:** Distribution of functional groups calculated on the basis of the deconvolution model of the XPS C 1s peak.

Sample Name	Total C 1s Peak Intensity (%)			
	C_1_	C_2_	C_3_	C_4_
Lipase	-	42	36	22
Chitin-lignin	9	25	46	20
Chitin-lignin + lipase	6	32	39	23

Since lipase contains a relatively small number of aromatic rings, originating from amino acids such as phenylalanine or tyrosine [55], the component C_1_ is not considered in the deconvolution of the C 1s spectrum for that substance. Component C_2_ prevails in the XPS signal, followed by C_3_. The support material is a mixture of chitin and lignin. The expected component ratio for pure chitin is C_2_:C_3_:C_4_ = 25:50:25 [59], while the ratio (C_1_ + C_2_):C_3_:C_4_ observed for lignin is 65:29:3 [60]. On the surface of the chitin–lignin matrix observed here, the contributions of components C_1_ and C_2_ are lower than would be given by a simple average for the mixture of chitin and lignin. Therefore, as suggested earlier, it is concluded that chitin prevails on the surface of the support. Comparison of the spectra of the chitin-lignin material and the product following enzyme immobilization indicates that C_1_ diminishes slightly, while C_2_ increases. Since C_2_ is dominant in the XPS spectrum of lipase, we believe this to be an indication of successful enzyme immobilization.

**Figure 4:**
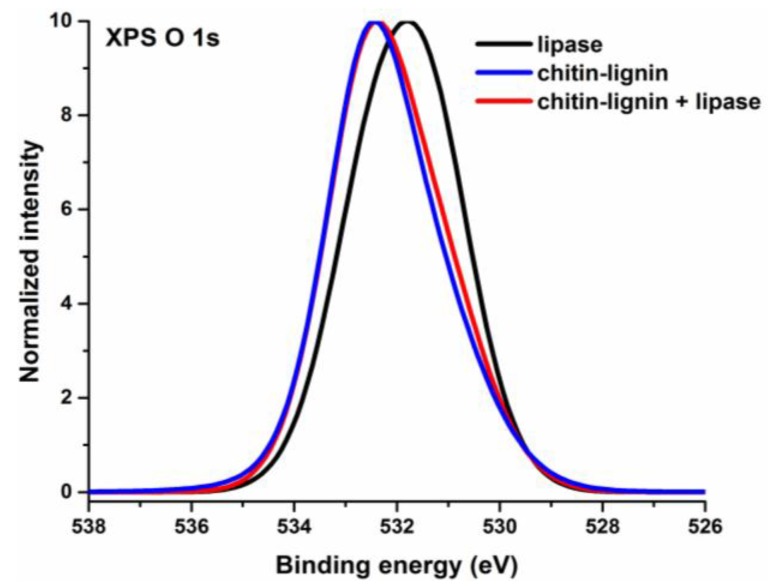
XPS O 1s spectra for lipase, chitin-lignin matrix and the product following enzyme immobilization.

Some additional evidence of the successful immobilization of lipase on the chitin-lignin matrix can be observed in the XPS O 1s spectra shown in Figure 4. The XPS O 1s transition observed for lipase is symmetric, with a maximum at binding energy 531.8 eV (dotted curve). In the case of the chitin-lignin matrix and the product of enzyme immobilization, the maximum of the O 1s peak is shifted in the direction of high binding energy to 532.4 eV. The structure of both chitin and lignin is dominated by C-OH groups, while in the case of the lipase a more equal ratio between hydroxyl and carboxyl groups is expected. The characteristic position of the O 1s peak for C-OH groups is approximately 532.5 eV, while its position for C=O groups is reported to be about 531.3 eV [61]. Accordingly, a shift in the XPS O 1s spectra is observed between the lipase and chitin-lignin. A small difference is also observed between the profile of the O 1s peak for chitin-lignin and for the chitin-lignin + lipase product. On the high-energy side of the spectrum the intensity of the O 1s peak obtained for the product following enzyme immobilization is slightly higher than the intensity of the peak obtained for the chitin-lignin support. The difference is small, but considering the relatively low quantity of immobilized lipase, it can be taken as confirmation of the increased concentration of C=O groups, which is an expected result of lipase being attached to the support.

XPS analysis provides no direct confirmation of lipase immobilization, since there is no apparent evidence of the formation of a new chemical environment. However, the formation of hydrogen bonds is not excluded. Moreover, the increase in the nitrogen-carbon ratio in combination with the subtle changes in the C 1s and O 1s component ratios can be considered an indication of successful immobilization of the enzyme.

#### 2.1.5. Electrokinetic Characteristic

**Figure 5:**
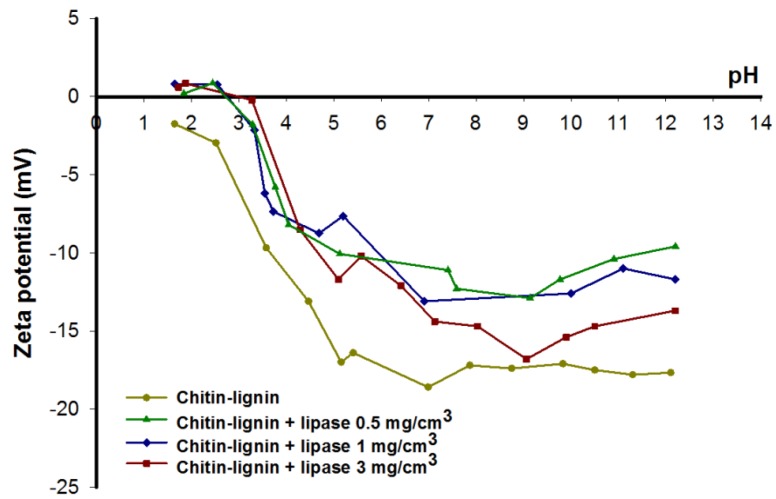
The zeta potential, as a function of pH, of the chitin-lignin material and selected products following immobilization.

Studies of zeta potential and the effect of pH provide very valuable data about the electrokinetic properties of dispersed systems. Figure 5 shows the results obtained. Determination of the zeta potential of the biocomposite with and without immobilized enzyme provides indirect confirmation of the effectiveness of the suggested method of immobilization. The graph shows the values of the zeta potential obtained for selected samples following immobilization for 24 h.

The zeta potential of the chitin–lignin system is negative over the whole of the investigated pH range, and the isoelectric point is not attained. This results from the presence of specific functional groups (-COOH and -OH) on the surface of the component biopolymers. The electrokinetic potential of pure kraft lignin is even more negative; its value increased when the lignin was combined with chitin (due to the presence of surface NH_2_ functional groups, which in an acidic environment can undergo protonation to NH_3_^+^) [53]. Lipase consists of several amino acids. The high percentage of acidic amino acids (Asp and Glu) gives the molecule a net negative charge, which is higher than the total for the positively charged residues (Arg, Lys, and His) [62]. That is why the isoelectric point of this protein is about 4 [63,64]. This value indicates that only at pH values below it will the surface charge (and indirectly zeta potential) be positive. The absolute value of zeta potential of chitin-lignin + lipase is smaller than this for matrix, especially in acidic condition, which can be explained by adsorption of lipase.

Following immobilization of the enzyme on the surface of the support, as a result of interactions between the surface groups of the support and of the enzyme, the absolute values of the zeta potential decreased. This provides indirect evidence of the adsorptive nature of the attachment of the enzyme to the chitin–lignin support [65,66,67]. There was a decrease in the number of the free functional groups which are responsible for generating the charge. In addition, the chitin-lignin products upon addition of enzyme attain their isoelectric point (the pH at which the zeta potential is zero), which had previously not been observed. From the measured values of zeta potential it can be concluded that the quantity of immobilized enzyme influences its electrokinetic properties [68]. Nevertheless, irrespective of the quantity of adsorbed enzyme, the value of the isoelectric point is 2.7.

#### 2.1.6. Quantity of Immobilized Enzyme

Based on the Bradford method [69] it was determined how the quantity of enzyme immobilized on the surface of the chitin-lignin support is affected by the concentration of the solution used in the immobilization process, and by the duration of the process. Table 5 contains detailed data on the quantity of biocatalyst adsorbed, depending on the concentration of the protein solution and the time of the process. The results are presented in terms of milligrams of enzyme per 1 gram of used matrix.

The results show that increasing the time of the immobilization process causes greater quantities of enzyme to be adsorbed. It should nonetheless be noted that the greatest increase in adsorbed protein occurs in the initial stages of the process. After the process time exceeds 4 h, the quantity of immobilized biocatalyst does not increase significantly, and the maximum change, depending on the concentration of the enzyme solution, is approximately 2 mg/g.

**Table 5:** Content of investigated elements in the chitin-lignin matrix and in the products following immobilization.

Immobilization Time	Concentration of Enzyme Solution (mg/cm^3^)		
	0.5	1	3
	Amount of Immobilized Enzyme (mg/g)		
**1 min**	1.45	5.13	6.19
**1 h**	6.23	9.76	14.97
**2 h**	8.17	10.84	18.46
**4 h**	8.58	11.37	18.72
**24 h**	9.22	11.84	19.31
**96 h**	9.94	12.57	20.28

Another parameter having a significant effect on the quantity of protein in the products following immobilization is the concentration of the solution used. The results show that the greatest quantity of protein is adsorbed from the solution with a concentration of 3 mg/cm^3^. When identical times of immobilization are compared, this solution enables the adsorption of more than twice as much protein as when a solution of concentration 0.5 mg/cm^3^ is used.

The greatest quantity of the enzyme was adsorbed from the solution with a concentration of 3 mg/cm^3^ following a process lasting 96 h. However, the optimum time of the immobilization process is 4 h, enabling comparable quantities of protein to be immobilized in a much shorter time, which has a positive impact on the economics of the studied process.

### 2.2. Hydrolytic Activity

#### 2.2.1. Determination of Hydrolytic Activity

**Figure 6:**
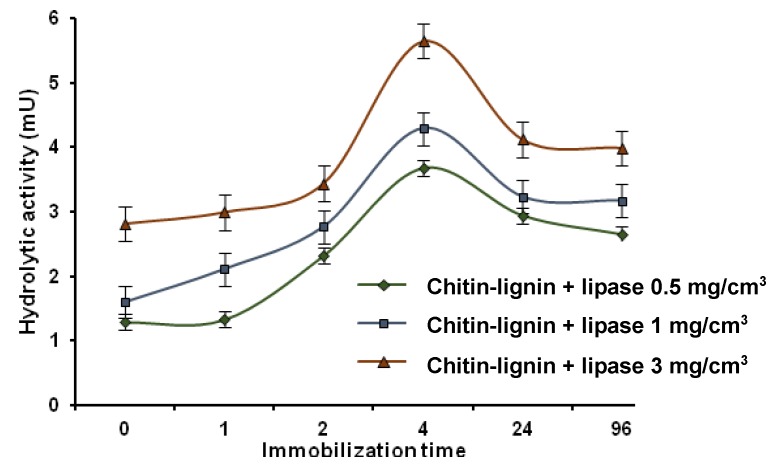
Graph showing changes in the catalytic activity of products depending on the time of immobilization and the concentration of the enzyme solution.

The hydrolytic activity of the free and immobilized enzyme was assessed spectrophotometrically based on the hydrolysis reaction of para-nitrophenyl palmitate. Figure 6 shows the results for catalytic activity of preparations with immobilized lipase obtained using enzyme solutions with concentrations of 0.5, 1 and 3 mg/cm^3^, subjected to immobilization over different time intervals. The measurements were performed at 30 °C.

The systems with immobilized enzymes have lower catalytic activity than the native lipase, for which the activity is measured at 7.46 mU. Irrespective of the concentration of the protein solution, the greatest activity is found for the products formed after 4 h of immobilization. The results showed the enzyme solution with a concentration of 3 mg/cm^3^ to be optimum for immobilization on a chitin–lignin support. The resulting immobilized lipase has the highest activity of all of the systems investigated, equal to 5.76 mU. This sample was selected for further analysis to determine the stability of the resulting system depending on the conditions of the catalyzed reaction.

The results show unambiguously that a greater quantity of immobilized enzyme does not lead directly to an increase in the system’s catalytic activity. The products obtained following 96 h of immobilization, which have the greatest quantities of immobilized protein, exhibit a lower activity. This is caused by the accumulation of too great a quantity of the enzyme on the matrix surface, blocking the active sites on the biocatalyst and thus reducing its activity [70].

#### 2.2.2. Thermal Stability

**Figure 7:**
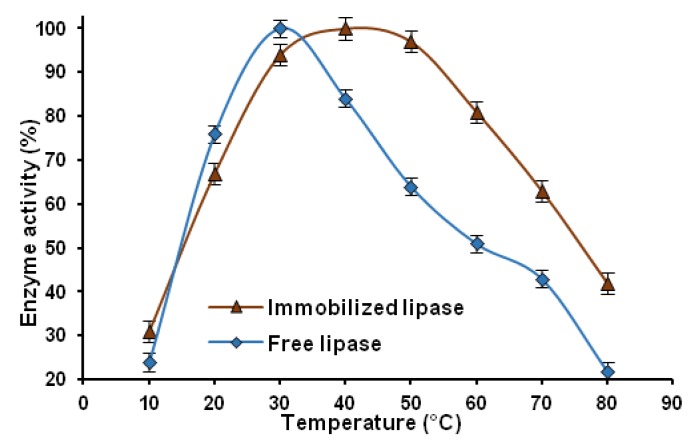
Graph of thermal stability of immobilized and native lipase in the temperature range 10–80 °C.

Thermal stability is one of the most important properties of immobilized enzymes. The thermal stability of the immobilized lipase was studied, in comparison with the native enzyme, over a temperature range of 10–80 °C. For this analysis, the system selected was one that underwent 4 h immobilization in the enzyme solution at a concentration of 3 mg/cm^3^ in phosphate buffer at pH = 7. Figure 7 shows a comparison of the thermal stability of the native lipase with that of the lipase immobilized on a chitin-lignin matrix.

The native lipase attains its maximum hydrolytic activity at 30 °C, while that of the immobilized enzyme occurs at 40 °C. It should be noted, however, that the immobilized lipase retains more than 90% of its initial activity even at 50 °C, where the properties of the free enzyme are lost to a significant degree. These results show clearly that attaching the biocatalyst to a solid support has a positive effect on its resistance to denaturation at high temperature. This has been shown to be a result of an increase in the rigidity of the protein structure [71]. The thermal stability increased because the immobilization process could protect the tertiary structure of the peptide from conformational changes caused by the higher temperature [72].

#### 2.2.3. pH Stability

**Figure 8:**
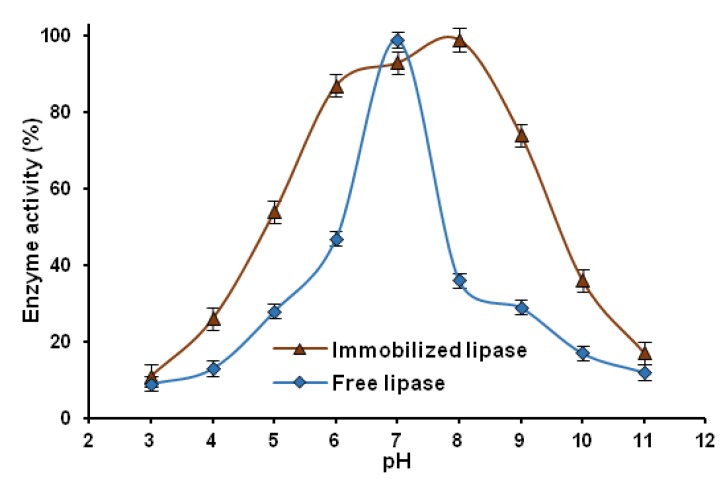
Graph showing changes in the catalytic active of immobilized and native lipase over the pH range 3–11.

The pH stability is an important characteristic of systems resulting from immobilization. The pH stability of the immobilized lipase, compared with that of the native enzyme, was studied over a pH range of 3 to 11 at 30 °C. Figure 8 shows a comparison of the pH stability of the native lipase with that of the lipase immobilized on a chitin-lignin matrix.

The data above show that the pH has a large effect on the activity of the lipase in an aqueous environment. The activity of native lipase reaches a maximum at pH = 7, and small changes in pH cause a large decrease in hydrolytic activity, by as much as 50%. The immobilized lipase has its highest activity at pH = 8, which is characteristic of immobilized enzymes in this catalytic group [73]. The attachment of the enzyme to a solid support also causes it to retain more than 70% of its activity in the pH range 6–9. The improved stability of the immobilized enzyme compared with the native protein is probably a result of conformational changes taking place in the protein tertiary and quaternary structure following immobilization [74]. An increase in pH stability of the immobilized lipase is also connected with the changes in spatial orientation of secondary structure of the protein backbone, caused by the formation of hydrogen bonds between the enzyme and matrix [75].

#### 2.2.4. Reusability

**Figure 9:**
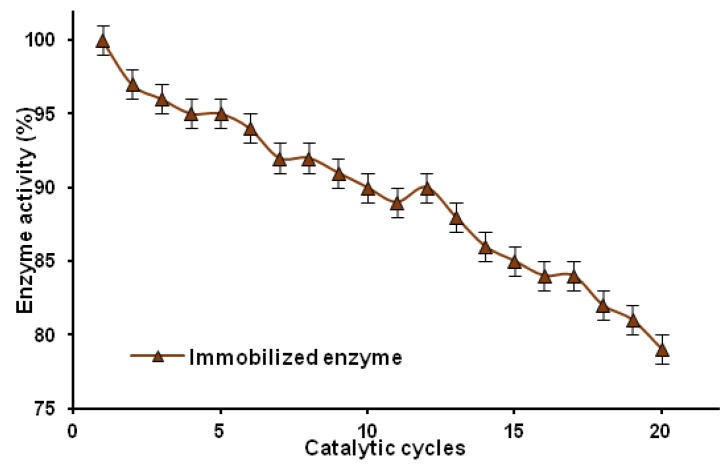
Changes in catalytic activity of immobilized lipase over 20 catalytic cycles.

Figure 9 shows the reusability of the lipase immobilized on the chitin-lignin matrix over 20 cycles. In each cycle, the immobilized lipase was separated and washed with phosphate buffer, and the activity was calculated for p-NPP hydrolysis.

The immobilized lipase was tested over 20 catalytic cycles, and was found to retain approximately 80% of its initial activity. The high reusability of products based on a chitin-lignin matrix may also lead to widespread use of this support in the immobilization of enzymes of other catalytic groups. Prolongation of the catalytic activity of these products may also lead to a significant reduction in the costs of carrying out reactions in real-life applications.

## 3. Experimental Section

### 3.1. Materials

The precursors, α-chitin powder from crab shells (technical grade) and kraft lignin (reagent grade), and 15% hydrogen peroxide as an oxidizing agent, were obtained from Sigma-Aldrich (Munich, Germany). Immobilization was carried out using commercial lipase from *Aspergillus niger* (Sigma-Aldrich, Munich, Germany) and phosphate buffer at pH = 7 (Amresco, Solon, OH, USA). The 85% phosphoric acid and 96% ethyl alcohol used in the Bradford method were obtained from Chempur (Piekary Śląskie, Poland). Coomassie Brilliant Blue G-250 (CBB G-250) was obtained from Sigma-Aldrich (Munich, Germany). The catalytic activity tests used para-nitrophenyl palmitate, Triton X-100 and gum arabic from Sigma-Aldrich (Munich, Germany) and 2-propanol from Chempur (Piekary Śląskie, Poland).

### 3.2. Preparation of Chitin-Lignin Material

The process of obtaining the chitin–lignin material (precursors ratio 1:1, *m/m*) began with the addition of 15 cm^3^ of 15% hydrogen peroxide to the lignin, according to the procedure reported in previously published work [53]. The mixture was subjected to intensive mixing at approximately 800 rpm for about 30 min using a high-speed stirrer (Eurostar Digital, IKA Werke GmbH, Staufen, Germany). Chitin was then added to the reactor, and mixing continued for 60 min. The resulting chitin-lignin material was filtered under reduced pressure and washed with distilled water. The product was then dried in a convectional dryer (Memmert, Munich, Germany) at approximately 105 °C for about 24 h.

### 3.3. Enzyme Immobilization

The process of immobilization of lipase from *Aspergillus niger* on the surface of the chitin-lignin composite was carried out using solutions of the enzyme at concentrations of 0.5, 1 and 3 mg/cm^3^ in a phosphate buffer at pH = 7, for times of 1 min and 1, 2, 4, 24 and 96 h. Quantities of 250 mg of the previously obtained matrix were placed in conical flasks, and 15 cm^3^ of the solution of the enzyme in the required concentration was added. The mixture was placed in a KS260 BASIC shaker (IKA Werke GmbH, Staufen, Germany), and shaken for the required length of time. Afterwards the precipitate was filtered under reduced pressure and left to dry at room temperature for 24 h.

### 3.4. Physicochemical Evaluation

The presence of the expected functional groups was confirmed by Fourier transform infrared (FTIR) spectroscopy, using a Vertex 70 spectrophotometer (Bruker, Karlsruhe, Germany). The materials were analyzed in the form of tablets, made by placing a mixture of anhydrous KBr (*ca.* 0.25 g) and 1.5 mg of the tested substance in a steel ring under a pressure of 10 MPa. The tests were performed at a resolution of 0.5 cm^−1^ in the wavenumber range 4000–400 cm^−1^.

^13^C CP MAS NMR measurement was carried out on a DSX spectrometer (Bruker, Karlsruhe, Germany). For the determination of NMR spectra, a sample of approximately 100 mg was placed in a ZrO_2_ rotator with diameter 4 mm, which enabled spinning of the sample. Centrifugation at the magic angle was performed at a spinning frequency of 8 kHz. The ^13^C CP MAS NMR spectra were recorded at 100.63 MHz in a standard 4 mm MAS probe using single pulse excitation with high power proton decoupling (pulse repetition 10 s, spinning speed 8 kHz).

The elemental contents of the chitin-lignin hybrid material and the immobilized enzyme were determined using a Vario EL Cube instrument (Elementar Analysensysteme GmbH, Hanau, Germany), which is capable of registering the percentage content of carbon, hydrogen, nitrogen and sulfur in samples after high-temperature combustion. A properly weighed sample was placed in an 80-position autosampler and subjected to combustion. The decomposed sample was transferred in a stream of inert gas into an adsorption column. The results are given to ±0.01%, and each is obtained by averaging three measurements.

The X-ray photoelectron spectra were obtained using Al *Kα* (hν = 1486.6 eV) radiation with a Prevac system equipped with a Scienta SES 2002 (VG Scienta, Uppsala, Sweden) electron energy analyzer operating at constant transmission energy (E_p_ = 50 eV). The spectrometer was calibrated using the following photoemission lines (with reference to the Fermi level): EB Cu 2p_3/2_ = 932.8 eV, EB Ag 3d_5/2_ = 368.3 eV, EB Au 4f_7/2_ = 84.0 eV. The instrumental resolution, as evaluated by the full width at half maximum (FWHM) of the Ag 3d_5/2_ peak, was 1.0 eV. The samples were placed loose in a grooved molybdenum sample holder. The analysis chamber was evacuated during the experiments to better than 1·10^−9^ mbar. Data processing involved background subtraction by means of an “S-type” integral profile and a curve-fitting procedure (a mixed Gaussian–Lorentzian function was employed) based on a least-squares method (CasaXPS software). The experimental errors were estimated to be ±0.1 eV for the photoelectron peaks of carbon and oxygen. Charging effects were corrected using the C 1s component attributed after deconvolution to aliphatic carbon bonds (component C_2_) and determined at 284.8 eV. The reproducibility of the peak position thus obtained was ±0.1 eV. The surface composition of the samples was obtained on the basis of the peak area intensities of the C 1s, O 1s, and N 1s transitions using the sensitivity factor approach and assuming homogeneous distribution of elements in the surface layer.

The electrokinetic stability of the materials with immobilized enzyme was determined on the basis of zeta potential dependence on pH, using a Zetasizer Nano ZS (Malvern Instruments Ltd., Worcestershire, UK) equipped with an autotitrator. Measurements were made in a 0.001 M NaCl solution over the pH range 2–12, using 0.001 M NaCl solution.

The quantity of immobilized enzyme was determined by the Bradford method [69]. A solution of the Bradford reagent was prepared by dissolving 10 mg of Coomassie Brilliant Blue G-250 in 5 cm^3^ of 96% ethyl alcohol, 15 cm^3^ of 85% phosphoric acid and 80 cm^3^ of water. In a quartz cuvette, 4 cm^3^ of the Bradford reagent was mixed with 800 µL of the analyzed protein solution and 100 µL water, and the analysis was performed 10 min after the preparation of the mixture. Measurements were made at wavelength 595 nm, using a JASCO 650 spectrophotometer (Jasco, Tokyo, Japan).

### 3.5. Evaluation of Hydrolytic Activity

The activity of the immobilized lipase was measured by the method used in our previous work [76], with slight modifications. Spectrophotometric measurements were made for 2 min at wavelength 410 nm at 30 °C, based on the transesterification reaction of para-nitrophenyl palmitate (p-NPP) to para-nitrophenyl (p-NP). Hydrolytic activity was measured in 1 cm^3^ quartz cuvettes containing 5 mg of immobilized lipase with 2.7 cm^3^ of substrate solution containing 10 mM phosphate buffer, 10 mM of p-NPP solution in 2-propanol, 0.44% mass fraction of Triton X-100 and 0.11% mass fraction of gum arabic. One mUnit of immobilized enzyme activity was defined as the release of 1 µmoL of p-NP per minute.

#### 3.5.1. Thermal Stability

The thermal stability of the immobilized and native lipase was determined over a temperature range of 10–80 °C. Hydrolytic activity was calculated as described in Section 3.5.

#### 3.5.2. pH Stability

The pH stability of the immobilized and native lipase was determined by incubating the substrate solution at different pH values (3, 5, 7, 9, 11) to compare the activity of the free and immobilized lipase. Catalytic activity was calculated as described in Section 3.5.

#### 3.5.3. Reusability

The reusability of the immobilized lipase was determined by testing over 20 cycles. Between each reaction step, the chitin-lignin matrix with the immobilized enzyme was separated from the substrate solution by centrifugation and washed with phosphate buffer. The hydrolytic activity was calculated as described in Section 3.5.

## 4. Conclusions

In this study, a chitin-lignin system was used as an innovative matrix in the process of immobilizing lipase from *Aspergillus niger.* Detailed characteristics of the obtained matrix, and confirmation of the effective immobilization of the enzyme, were obtained using such techniques as FTIR, XPS, ^13^C CP MAS NMR and elemental analysis. It was shown that both the time of the process and the initial concentration of the protein solution have a significant effect on the properties of the products obtained. A determination was also made of the quantity of enzyme immobilized on the surface of the system, and of the catalytic activity of the system following lipase immobilization. It was found that the immobilized lipase exhibits lower activity than the free enzyme, but retains its catalytic properties for a greater number of reaction cycles. The enzyme bound to the chitin-lignin matrix also has greater thermal and chemical stability than the native protein. Measurement of the zeta potential enabled determination of the electrokinetic properties of the systems obtained. Detailed analysis of the FTIR spectra of the products of the immobilization process, and changes in the zeta potential and shifts in signal maxima on ^13^C CP MAS NMR spectra, indicate that the enzyme is attached by way of physical adsorption, probably through the formation of hydrogen bonds.
